# A Patient and Public Involvement and Engagement Approach to Co-Design a Walking Football Intervention for People with Chronic Breathlessness

**DOI:** 10.3390/healthcare14203515

**Published:** 2026-10-19

**Authors:** Callum Bradford, Sophie Suri, Kirsti J. Loughran, Denis Martin, Noelle Robertson, James Edwards, Samantha L. Harrison

**Affiliations:** 1 School of Health and Life Sciences, Teesside University, Middlesbrough TS1 3BX, UK; callumbradford@wirral.gov.uk (C.B.); s.suri@tees.ac.uk (S.S.); k.loughran@tees.ac.uk (K.J.L.); d.martin@tees.ac.uk (D.M.); 2 National Institute for Health and Care Research (NIHR), Applied Research Collaboration (ARC), Northeast and North Cumbria (NENC), Newcastle Upon Tyne NE3 3XT, UK; 3 School of Psychology and Vision Sciences, University of Leicester, Leicester LE1 7RH, UK; nr6@leicester.ac.uk; 4 Breathe Easy Darlington, Darlington DL1 1LJ, UK

**Keywords:** chronic breathlessness, pulmonary rehabilitation, walking football, physical activity, patient and public involvement and engagement, co-design

## Abstract

**Highlights:**

**What are the main findings?**

• Novel, active PPIE approaches, including involvement in walking football (WF) taster sessions, enabled people with chronic breathlessness to meaningfully co-design WF for this population group.
• PPIE members identified important barriers to participation in WF, including keeping pace with others, balance/turning, oxygen/walking aid usage and travel challenges, and generated practical adaptations.

**What are the implications of the main findings?**

• Active experiential co-design approaches can be conducted safely and ethically and may generate richer insights than discussion-based methods alone.
• Building trust with PPIE members and allowing sufficient flexibility for iterative changes is essential to develop interventions tailored to the needs of the population.

**Abstract:**

Background: Walking football (WF) was identified as a potential solution for exercise maintenance after pulmonary rehabilitation. However, WF has not previously been made available to people with chronic breathlessness. This work undertook a novel approach to Patient and Public Involvement and Engagement (PPIE) in which PPIE members, recruited from Breathe Easy Darlington (a charity), helped to co-design a WF intervention for people with chronic breathlessness. Methods: PPIE activities included: an introductory stakeholder meeting (*n* = 20) to discuss WF, four ‘WF sessions’ (*n* = 10–12), and a debrief following the first WF taster session (*n* = 12). Results: PPIE members listed several concerns at the introductory meeting, namely: (1) keeping pace with non-breathlessness people; (2) limited balance/turning ability; (3) requirement of walking aids/oxygen; and (4) accessible parking and low-light driving conditions. Responding to these concerns, WF taster sessions were: (1) made available exclusively to people with breathlessness with a “buddy system” employed for new players; (2) no-contact “table football-style” rules and a weighted “slow-motion” football were applied; (3) oxygen was worn on the person and walking aids could not make contact with the ball; and (4) accessible parking was abundant and sessions concluded by 2pm. Lessons learned included having trust in your PPIE members, that active forms of PPIE can work successfully when conducted in an ethical manner and when working together with PPIE members, it is important to find balance regarding researcher involvement in activities. Conclusions: PPIE led to important changes that improved the research project and intervention design. WF would not have been viable for people with chronic breathlessness without these iterative adjustments.

## 1. Introduction

Patient and Public Involvement and Engagement (PPIE) refers to research that is carried out *‘with’* members of the public rather than *‘to’* or *‘for’* them [1,2]. Examples include involving members of the public to analyse data, or as co-applicants for research funding. Such approaches to research provide opportunities for people with unique perspectives and experiences to have a greater influence upon research design, rather than simply providing feedback for an intervention or finished project [2].

Public involvement methods have been of particular interest to researchers concerned with chronic breathlessness. This is because people with chronic breathlessness often find it difficult to express the complexity of their conditions through conventional research methods, finding it hard to describe their symptoms due to the ‘invisibility’ of the illness [3]. Further, talking about breathlessness can be emotionally challenging, increasing anxiety, which can make breathing and speaking more difficult [4]. People with chronic breathlessness may also feel a sense of illegitimacy and discomfort in a research context [4]. Public involvement approaches that utilise more creative methods of engagement have proved successful, with one known example utilising comic-based art workshops, providing the opportunity to communicate in a more creative and effective way than would be available in a typical interview format [4].

Chronic breathlessness is a major cause of ill health and disability worldwide, leading to significant human and economic burden [5,6]. Pulmonary rehabilitation (PR), a combination of supervised exercise and education, is recommended for people with chronic breathlessness [5]. However, many of the benefits gained often diminish within 6–12 months of completion [6,7]. This paper aimed to develop an ‘exercise maintenance’ intervention, which those who complete their PR programmes could progress onto and exercise sustainably. This exercise maintenance intervention would be based upon WF, which shares similar rules to association football, but with no running, less physical contact, and often on a smaller scale. WF has shown promise among populations including individuals with prostate cancer, diabetes and dementia [8]. However, in developing this intervention, the researchers came across a barrier, notably that WF had not previously been delivered to patients with chronic breathlessness, and therefore it would be difficult for people without prior exposure to an activity to comment meaningfully on its feasibility.

Novel PPIE methods were therefore needed so people with chronic breathlessness could co-design the WF intervention. This paper describes these applied methods, which are referred to as trial ‘taster sessions’ of WF, providing the opportunity to design a WF intervention *with*, as opposed to *for*, people with chronic breathlessness. It also reflects on the lessons learnt from the PPIE process, that not only informed delivery and adaptations to the intervention but also how the PPIE activities themselves were delivered.

## 2. Materials and Methods

### 2.1. Approach to Public Involvement

The UK standards for public involvement [9] and NIHR Community Engagement Toolkit [10] were consulted throughout the PPIE process. Reporting of the PPIE was guided by the GRIPP2 checklist [1]. Detailed reflective notes were recorded during and after the PPIE sessions. Following each session, the notes were reviewed by the research team (CB, SLH, KL, SS) and key points relevant to the development of the WF intervention were identified and summarised. The information was subsequently consolidated into Table 1, Table 2 and Table 3 to provide an overview of the feedback provided by PPIE members and how this informed intervention development. The public involvement approach was implemented on a co-design basis with PPIE members from Breathe Easy Darlington. Breathe Easy Darlington is a charity for people with pulmonary diseases (as well as family and carers), offering people who struggle with breathlessness the opportunity to share experiences and stories with others living with lung conditions. Co-design refers to collaboration between researchers, designers, developers, and service users who are considered knowledgeable in their respective experiences [11,12]. When a patient or member of the public contributes towards the planning, design, or implementation of an intervention, it is co-designed [13]. However, this contribution must be more than observing or consulting with patients; rather, it should be a joint effort to explore needs and potential solutions for an intervention [12,14]. When a service is developed on a co-design basis, there is a greater likelihood that it will align with patient needs and preferences, creating more efficacious and sustainable interventions [15]. The co-design of the WF intervention was conducted via a two-phase approach—an introductory meeting (phase one) and WF taster sessions (phase two).

### 2.2. Ethics and Safety

Ethical approval was not required for this PPIE project, as per NIHR guidelines, as members of the public worked collaboratively with the research team to inform the development of the WF intervention, with no personal or research data collected from contributors [2]. Nevertheless, ethical good practice was followed, including obtaining informed consent for the use of members’ images [16].

Safety was considered throughout the PPIE process. Before formal PPIE activities, the researchers consulted the scientific literature and relevant healthcare professionals and conducted fieldwork with three stakeholder groups: pulmonary physiotherapists during pulmonary rehabilitation, a WF Association representative with international coaching experience, and active WF players. This highlighted several safety considerations, including the need for self-paced activity, accessible indoor venues, and specialised coaching, as the pace of conventional WF exceeded initial expectations.

Once PPIE members commenced the taster sessions, a risk assessment was undertaken and reviewed following each session. Following a collision between two players, additional safety measures were introduced, including research team members undertaking emergency first aid training, implementation of a pre-session safety checklist, trialling alternative footballs, and modifying the rules to prohibit physical contact. Members were also asked to provide relevant health information, including previous injuries, inhaler requirements and emergency contacts. An experienced WF coach was present throughout to teach appropriate techniques and monitor the pace of play. Information sheets and image consent forms were also provided to ensure members understood their role in intervention development and consented to the use of photographs for dissemination.

### 2.3. Co-Design Phase One—Introductory Meeting

Phase one of the co-design involved introducing the concept of WF to 20 PPIE members from Breathe Easy Darlington (March 2022). To introduce the idea, the researchers attended one of Breathe Easy Darlington’s monthly social catch-up meetings. The researchers decided to travel to the PPIE members (rather than invite them to the university campus or an alternative meeting place) as per Community Engagement guidelines [10]. The engagement toolkit suggests researchers should, where possible, “fit yourself around others and be willing to meet in community settings” and “be extremely mindful of any accessibility issues”. Accessibility is unfortunately a common hurdle people with breathlessness are expected to overcome. Therefore, had the researchers invited them to a university campus where the parking is highly limited, many PPIE members would have felt unable to attend.

### 2.4. Co-Design Phase Two—WF Taster Sessions

Following the introductory meeting, the researchers invited PPIE members to partake in four ‘taster sessions’ of WF to develop the intervention, with a core group of 10–12 members attending each session (July to October 2022) (age 55–75 yrs; 3 males; 2 oxygen users, 1 person required a walking aid; respiratory conditions included COPD, asthma, bronchiectasis and type 2 respiratory failure). The reduction in numbers following the introductory meeting was due to the distance of the sports hall from where PPIE members usually met. After the first session, the researchers also met again with 12 PPIE members for an informal ‘debrief’ to further evaluate the session. WF sessions were 2 h each (plus time set aside to socialise/reflect over tea/coffee afterwards), and consisted of a ten-min warm-up, passing/dribbling drills, small-sided matches, and fun mini-games drawing from other sports (e.g., tennis, netball, hockey). Sessions were administered by an experienced WF coach with assistance from the researchers.

## 3. Results

### 3.1. Co-Design Phase One—Introductory Meeting

Despite the researchers’ previous research and fieldwork with pulmonary and WF professionals, the PPIE members were very sceptical during the introductory meeting that people with chronic breathlessness would be capable of playing WF to a capacity that would be perceived as enjoyable. So, the researchers suggested a proposition. PPIE members would describe any concerns and apprehensions they had regarding WF, and together, the researchers would work to create solutions so they would potentially feel comfortable playing. These concerns and related solutions are outlined in Table 1.

**Table 1:** Concerns raised during introductory PPIE meeting.

Concerns and Apprehensions	Applied Changes
Worried they would not be able to play due to requiring walking aid and/or oxygen therapy.	• Those who required walking aids or needed to carry oxygen would not be excluded from the intervention, and the WF would be adapted as needed to cater for this (e.g., oxygen carried in a backpack).
Concerns they lacked the exercise capacity, balance, and turning ability required to play football to an enjoyable and comfortable standard with people who do not have pulmonary conditions.	• WF was made exclusive to people with chronic breathlessness. This reassured PPIE members that the football would be competitive, and not a ‘non-contest’ between themselves and experienced non-breathlessness players.
Limited previous experience with football, lacked confidence in understanding the rules and implementing them consistently, especially if playing alongside experienced players.	• The WF coach understood that most of the PPIE group had little prior experience with playing football, and therefore adapted the intervention, introducing the rules and skill work slowly as they progressed.
Concerns if the weather is sub-optimal, the football would not be enjoyable or enhance symptoms.	• The WF was set in an indoor sports hall.
Accessibility and parking.	• The WF venue was chosen due to having plenty of free accessible parking very close to the entrance, with no stairs and an accessible lift.
Travelling to/from the venue in the late evening once it becomes dark outside.	• All sessions finish by 2pm, so PPIE members have time to travel home before it becomes dark, especially in the winter months.
Limited time for breaks when needed.	• Sessions were planned with regular break times throughout. Drills and games are designed so PPIE members can sit down and take a break easily (i.e., putting seating around the pitch) at any time without needing to inform the researchers. The researchers would then check on people sitting out as needed.

### 3.2. Co-Design Phase Two—WF Taster Sessions

Due to the concerns previously raised in the introductory meeting, PPIE members remained sceptical and were somewhat hesitant to play in the WF taster sessions. However, as the researchers had formed a long-standing and mutually beneficial relationship with the PPIE members via various PPIE activities [4], they trusted that the researchers would not ask them to partake in anything that was overly hazardous or uninteresting. Working together and developing these trusted relationships (particularly with a respected leader of the group who can encourage others) are key to public involvement in research [9] and proved especially beneficial to the development of the intervention with each subsequent WF session, as described in Table 2, which documents the feedback and reflections from each taster session.

It is important to take note of the incident that occurred in session 3, and how the researchers later adapted for this in session 4. PPIE does not typically require ethical clearance, but it still needs to be conducted in an ethical manner, e.g., using image consent forms, pre-exercise screening, first aid training, gaining emergency contact details, and secure data storage. Because the researchers took these measures prior to play, they were able to respond to the incident quickly and appropriately.

**Table 2:** Post taster session feedback and reflections with PPIE group.

Taster Session	Feedback and Reflection
Taster Session 1 (including a debrief meeting with PPIE group)	Feedback from the PPIE group was very positive. The applied precautions, such as having chairs around the outside of the pitch for people to catch their breath when needed, or planning for extended/frequent break times, were appreciated. The warm-up was created into a small competitive game between two sides, which added to the enjoyment of the sessions, with PPIE members stating that the sessions distracted them from their symptoms, allowing them to exercise more than in different contexts. During the debrief, PPIE group members stated that they “surprised ourselves”, and “on the pitch you forget about breathlessness”, telling new members, “It’s amazing, give it a try, it’s a laugh!”. Time was set aside after the sessions to socialise and reflect over tea/coffee. This element was acknowledged as increasing the enjoyability of the sessions and perceived as fundamental to the intervention’s longevity. Involvement elicited feelings of nostalgia for “playground games” from school. One warm-up game involved throwing and catching tennis balls at a wall; several members of the group noted how they used to play the game at school, and they were happy they still had the muscle memory to play in a skilful manner.
Taster Session 2	This session ran slightly less smoothly and was described as not as enjoyable as the first for several reasons: • The session plan was not well kept to, in that breaks went on too long as the researchers were organising the next games or drills, and the team struggled to improvise as well after a late start. • The researchers were asked to help make up the numbers for certain drills but became overly involved in the intervention, making it less enjoyable for the PPIE group. • A practice drill was tried using chairs as goals to encourage movement and passing, but the PPIE group found it confusing and overcomplicated, preferring to just play with the ‘proper’ goals.
Taster Session 3	The first part of the session ran as normal with a warm-up and practice drills. However, the session was stopped after an hour due to an accident with two players coming together going for the ball. Neither player was seriously injured. Recommendations to attend a medical check-up were declined.
Taster Session 4	After the previous session, further action was taken to reduce the level of risk and improve the researchers’ ability to respond to accidents when they occur. As a result, the researchers received positive feedback from the PPIE group, and the session flow was perceived as “smoother” as opposed to stop-starting. Changes applied include: • The researchers completed an emergency first aid course (the coach was already first aid-trained, and two members of the research team were qualified physiotherapists). • A pre-football checklist was designed to be signed off before each session to ensure the proper precautions had been taken (e.g., proper footwear, health information up to date). • A stricter approach was taken in reducing the pace of play, and a no-contact rule was applied to reduce the risk of accidents. • During drills and games, the competitive aspect was played down, reducing the pace of the game. The researchers also implemented a yoga-based warm-up to ‘ground’ players prior to playing. • The researchers experimented with different types of football to see if it could be used to reduce the pace of play (e.g., a weighted ball) and found that soft foam-style footballs were best and perceived to be most enjoyable to use.

### 3.3. The UK Standards for Public Involvement

Below in Table 3 are the UK standards for public involvement and engagement [9] mapped to the PPIE activities with the PPIE members, alongside reflections on areas for improvement. These standards were referred to throughout the development process not only to improve the quality of the work, but to build trust, transparency, impact, and foster collaboration between stakeholders, all within a respectful and systematic framework.

**Table 3:** PPIE approach mapped onto UK standards for public involvement.

UK Standard	Summary of Standard	Activity	Areas for Improvement
Inclusive opportunities	Offering public involvement opportunities that are accessible and reach people and groups according to community and research needs	WF was designed exclusively for people with chronic breathlessness.	The distance from where PPIE members were based to the sports hall was too far to travel long term (approx. 20 min drive/13 miles) and was only accessible to those who had a car/transport.
Working together	Work together in a way that values all contributions, and that builds and sustains mutually respectful and productive relationships	The researchers would occasionally participate in the WF with PPIE members to provide support, build rapport, and experience the game first-hand. The researchers valued the contributions PPIE members made, applying changes to the WF based on their comments.	Active participation could occasionally increase pace of play, despite the intervention being self-paced.
Support and learning	Offer and promote support and learning opportunities that build confidence and skills for public involvement in research	PPIE members developed football skills in an enjoyable “real-life” context, demonstrating balance skills (such as standing on one leg), which outside of the intervention they would not have the confidence to attempt. Training and resources were provided to enable PPIE members to participate in a meaningful way.	The researchers identified and addressed various risks, including limited balance/turning ability and the use of oxygen/walking devices. This should be improved upon in any future implementations to further understand and reduce the risk of injury, in turn increasing people’s confidence to participate in public involvement activities. For example, improved carrying equipment for oxygen tanks would likely increase safety and enjoyment.
Communication	Use plain language for well-timed and relevant communications as part of involvement plans and activities	Time is set aside at the end of all WF sessions to socialise over tea/coffee. This time is utilised between the researchers and PPIE members as an informal debrief of the previous session, and future planning.	It may have been beneficial to develop a more formal communication plan, so PPIE members have a clear waypoint if they would like to discuss any given issue.
Impact	Seek improvement by identifying and sharing the difference that public involvement makes to research	Transparent dissemination of the intervention development process.	To further understand the PPIE members’ experience of contributing to the design of a WF intervention.
Governance	Involve the public in research management, regulation, leadership and decision-making	All information shared on a public involvement basis is kept confidential until the researchers receive explicit permission to share it via publications, reports, and presentations. PPIE members were actively involved in decision-making regarding changes to the WF intervention.	Invite PPIE members to lead sessions alongside WF coach, so they have a more active role in development.

## 4. Discussion

### 4.1. Creative and Active PPIE Approaches Can Be Implemented Successfully

The iterative co-design of WF was only possible through the novel use of taster sessions—a physically active form of PPIE that required substantial resources, capacity, and preparation. Crucially, what the researchers have learnt is that active PPIE methods are not only feasible for people with chronic breathlessness, but mutually beneficial. From a research point of view, if the researchers had omitted phase two of the co-design and only conducted the more ‘traditional’ PPIE methods described in phase one, they likely would have deemed WF unfeasible for people with chronic breathlessness without the changes the PPIE members implemented. For the PPIE members, they benefited from a fun, confidence-building intervention personalised to their needs. They also sought further funding to continue the walking football on their own accord, without the involvement of the researchers (£10,000 National Lottery funding, July 2023). This is an example of how sharing power and decision-making can promote empowerment [17]. Empowering individuals to maintain exercise post-pulmonary rehabilitation is crucial as many of the benefits of pulmonary rehabilitation are not maintained long term [6,7].

### 4.2. PPIE Needs to Be Conducted in an Ethical Manner

PPIE activities do not typically require formal ethical approval [16], as members of the public are involved as collaborators within the research team rather than as research participants from whom data are collected. However, this does not mean that ethical considerations can be overlooked when undertaking PPIE, particularly where activities involve physical exercise and may therefore present an increased risk of injury [16]. In this work, ethical and safety considerations directly informed the development and refinement of the WF intervention, demonstrating that these issues should not be viewed as separate from intervention development but as an integral component of its design and delivery. The implementation of appropriate precautions, alongside iterative adaptations throughout the process, was essential to ensuring that public involvement remained safe, appropriate, and meaningful. This highlights the value of adopting an ‘ethical anchor’ when undertaking more active forms of PPIE, whereby ethical and safety considerations remain embedded throughout the research process and continue to inform decision-making as the intervention evolves.

### 4.3. Trust Your PPIE Group

One key point raised during the PPIE included making the WF exclusive to people with breathlessness. This contradicted previous advice received from WF players and associations, who recommended opening up the session to as many people as possible to promote sustainability. As this was the first and primary sticking point for the PPIE members when considering participating, the researchers decided to trust the PPIE group’s advice to make the walking football only for people with chronic breathlessness. This led to a substantial change in design not only for the intervention, but the research project. In the initial protocol, after PPIE members helped to design the intervention, the researchers had planned to slowly introduce patients from PR into an already ongoing local WF team. This team did not have rules regarding inclusion and was open to all who were interested. However, from this initial feedback from the PPIE members, it was quickly learned that the researchers would struggle to recruit in the future for a project that was not specifically tailored for people with chronic breathlessness. This one point is a great example of the importance of PPIE and building trusted relationships, as without consulting the PPIE members, the researchers likely would have stuck with the initial protocol, in turn generating poor recruitment and adherence rates and missing an opportunity to co-design a WF intervention for people with pulmonary conditions [18].

### 4.4. Lessons on Working Together

When involving PPIE members in the co-design process, the researchers would occasionally join in with the WF given the emphasis on developing the intervention together. ‘Working together’, a key pillar in the UK Standards for Public Involvement, gave the researchers greater insight into how WF worked, and the potential challenges people playing it may face [9]. This also helped set the social dynamic of working and enjoying WF together, rather than watching PPIE members from the sidelines as would be expected with a traditional researcher–participant dynamic. However, during certain football drills, the addition of the researchers upset the dynamic. The more researchers became involved, the less valid PPIE members’ evaluation of the intervention became, given it was no longer the sport they had envisioned. This was recognised and attended to early on in the process, but the lesson here is that it is important to find the right public–researcher balance, especially with more active PPIE methods, and just because you are designing an intervention together does not mean all activities need to be participated in equally by researchers and PPIE members alike.

### 4.5. Limitations

There are several limitations of the current work that should be acknowledged. Firstly, the PPIE members were self-selected from a single charity within one UK region, which may limit the transferability and generalisability of the findings to wider populations. The sample may also have been biased towards individuals who were able to travel to the study location, as the sports hall where the WF sessions were held was approximately a 20–30 min drive from where most members lived. Future work should consider providing transport options, such as taxis or a dedicated bus service, to reduce potential barriers to participation and facilitate involvement from a larger and more diverse group of PPIE members. Additionally, qualitative data were captured through reflective notes, which are inherently subject to selective recording and researcher subjectivity. Consequently, the findings should be interpreted with consideration of these potential sources of bias.

## 5. Conclusions

This paper outlines the utilisation of PPIE towards the co-design of a WF intervention created exclusively for patients with chronic breathlessness. The contribution of PPIE members from Breathe Easy Darlington through various PPIE activities proved invaluable to the development of the WF intervention, informing design choices such as making WF exclusive to people with breathlessness, providing adequate rest time between drills, and selecting an appropriate host venue, all of which were fundamental to the intervention’s feasibility. Lessons learned included trusting PPIE members, recognising that active forms of PPIE can work successfully, ensuring PPIE is conducted ethically, and finding the right balance of researcher involvement when working with PPIE members. Future work should look to adopt more innovative and active forms of PPIE.

## Data Availability

The original contributions presented in this study are included in the article. Further inquiries can be directed to the corresponding author.

## Abbreviations

The following abbreviations are used in this manuscript:

COPD	Chronic Obstructive Pulmonary Disease
PPIE	Patient and Public Involvement and Engagement
PR	Pulmonary Rehabilitation
WF	Walking Football
GRIPP2	Guidance for Reporting Involvement of Patients and the Public
